# Factors associated with nutritional status, knowledge and attitudes among tuberculosis patients receiving treatment in Ghana: A cross-sectional study in the Tema Metropolis

**DOI:** 10.1371/journal.pone.0258033

**Published:** 2021-10-14

**Authors:** Prince Kubi Appiah, Bright Osei, Hubert Amu

**Affiliations:** 1 Department of Family and Community Health, School of Public Health (Hohoe Campus), University of Health and Allied Sciences, Ho, Ghana; 2 Department of Medical Law and Ethics, Asian Institute for Bioethics and Health Law, College of Medicine, Yonsei University, Seoul, South Korea; 3 Department of Population and Behavioural Sciences, School of Public Health (Hohoe Campus), University of Health and Allied Sciences, Ho, Ghana; Federation University Australia, AUSTRALIA

## Abstract

**Background:**

Nutritional deficiencies are generally associated with increased risk and severity of tuberculosis. This study investigated the nutritional status, knowledge, and attitudes of tuberculosis (TB) patients receiving treatment in the Tema Metropolis.

**Method:**

A cross-sectional design was used to collect data on the nutritional knowledge, attitude, and status of TB patients. Nutritional status was analysed using World Health Organization’s formula for body mass index. Pearson’s chi-square and logistic regression models were used to assess associations between predictor and outcome variables. All statistical analyses were considered significant at p-values < 0.05.

**Result:**

The prevalence of malnutrition among TB patients was 39.7%, 14.4%, and 4.8% for underweight, overweight, and obesity respectively. There was a high (61.0%) knowledge of nutrition among the patients. Also, 65.8% had good attitude towards nutrition. There were significant associations between normal nutritional status and age of the TB patients (*p* = 0.041), highest educational level attained (*p* = 0.036), employment status (*p* = 0.019), status of alcohol intake (*p* = 0.031), number of months on TB treatment (*p* = 0.021), and attitude towards nutrition (*p* = 0.028).

**Conclusion:**

There was a reasonable nutrition-related knowledge and attitude towards nutrition among the TB patients. However, that did not reflect on their nutritional status. We recommend continuing education on smoking cessation, avoidance of harmful use of alcohol, and the establishment of food aid and other livelihood intervention programs for TB patients.

## 1. Introduction

Globally, an estimated 10 million people were infected with tuberculosis (TB) in 2018, as the disease continues to be a major public health concern in most developing countries, including Ghana [1]. The same reports indicated that 2 million four hundred and fifty thousand (2,450,000), and forty-four thousand (44,000) of the TB patients live in Africa and Ghana, respectively [1]. Malnutrition also persists at unacceptably high levels on a global scale. Among children under five years of age, 149.0 million, 49.5 million, and 40.1 million were stunted, wasted, and overweight, respectively. Also, there are 677.6 million obese adults (aged 18 years and over). The report indicated that 58.8 million (29.1%), 14.0 million (6.4%), 9.5 million (4.7%) of the children in Africa were stunted, wasted, and overweight, respectively, while an estimated 18.4% female and 7.8% male adults on the continent are living with obesity. The same data also revealed that about 17.5%, 6.8%, and 1.4% of Ghanaian children were stunted, wasted, and overweight, respectively. About 16.6 female and 4.5% male adults in Ghana are also living with obesity [2].

Tuberculosis usually comes with conditions like anorexia, cachexia, and generalized weakness leading to low dietary intake [3]. Malnutrition is, therefore, frequently observed in patients with the disease [3]. Research has shown that patients with active pulmonary tuberculosis are always malnourished with reductions in visceral proteins, anthropometric indexes, and micronutrient status [4], with sex, educational level, household income, and HIV/AIDS status as significant contributors to the nutritional status [5]. A study in Ghana, also reported that half of the newly diagnosed TB patients were moderately or severely malnourished at the time of starting their treatment [6].

Nutritional deficiencies are generally associated with increased risk and severity of tuberculosis by adversely affecting immunological mechanisms that are crucial for success in any control measures, including case management. However, improvements in nutritional status with the commencement of treatment have been demonstrated [7]. Although the link between malnutrition and TB has been established, there is not enough literature in Ghana in this area, especially after patients have started treatment [6]. Hence, the current study assessed the nutritional status, knowledge, and attitudes among TB patients receiving treatment at TB centres in the Tema Metropolis.

## 2. Materials and methods

### 2.1 Study site

This study was conducted in the Tema Metropolis. Tema Metropolis is one of the twenty-nine districts in the Greater Accra Region, located in the South-eastern part of Ghana [8]. It is a vibrant commercial and industrial city and has the main sea-port entry to Ghana. It is the second populated area in the Region, with an annual growth rate of 3.1 and an estimated population of 529,960 at the time of the study in 2019 based on the 2010 population of 402,637 [9]. Being the industrial and port city of the country comes with myriad of health implications for the population. For instance, HIV/AIDS, Tuberculosis, and Occupational Accidents are more prevalent than other parts of the country [10].

### 2.2 Study population

The study population comprised people aged 15–65 years old, residing in the Tema Metropolis, who had been diagnosed with tuberculosis and were receiving treatment.

### 2.3 Study design

An analytical cross-sectional design was used to collect data on nutritional status, knowledge, attitude, status among TB patients. The design was adopted for this study because it sought to explore the prevalence of nutritional status and other factors among TB patients who were receiving treatment in the Tema Metropolis.

### 2.4 Sample size and sampling procedure

A total of 146 TB patients receiving treatment in the metropolis were involved in the study. Census approach was adopted to recruit all those who were receiving treatment at the time of the data collection due to the relatively small number of the population.

### 2.5 Data collection tools and procedure

We interviewed and collected anthropometric data from tuberculosis patients who were receiving treatment after the processes involved in the study were explained to them. We used participants’ contact information obtained from them after attending sessions and were leaving TB treatment centres and trace them to their homes for the data collection. We adopted this approach to ensure that they are free from intimidation and fear from health providers and freely express themselves, as well as to reduce information bias.

We used a pre-tested semi-structured questionnaire to collect data including sex, age, ethnicity, religion, marital status, highest educational level, employment, smoking, alcohol intake, and months on treatment, as well as knowledge and attitude towards nutrition from the participants.

We use an electronic weighting-scale produced by SECA to take the weight measurement of participants. Their weights were measured without wearing shoes, heavy clothing, and holding on to any support. The weighting-scale was calibrated before starting each day’s work and repeated the calibration after weighing every five people. We used a non-stretchable tape (height measurement instrument called Microtoise) to take the height measurement of participants, without them wearing footwear, headgear, and hat/cap.

The data collection instrument we used was adapted from a similar study among People Living with HIV/AIDS (PLHIV) in Armenia. The tool consists of four sections (A-D); Section A was on socio-demographic characteristics, B was on nutrition-related knowledge and a healthy diet including dietary recommendations, source of nutrients, choosing everyday foods, and diet-disease relationships, C was on the attitude towards healthy nutrition, and D was on nutritional status of the participants [11]. Data collection was between September and October 2019.

### 2.6 Data analysis

We used Statistical Package for the Social Sciences (SPSS) version 22.0 to analysed the data into descriptive statistics such as mean, frequencies, and percentages and presented the information in tables and charts. Pearson’s chi-square and logistic regression models were used to assess associations between predictor and outcome variables, and *p*-values < 0.05 at 95% confidence interval was considered statistically significant.

We used twenty questions to evaluate participants’ knowledge of nutrition, and each correct response was rated 1 point, while a wrong response was rated zero. Participants’ overall nutritional knowledge was categorized using modified Bloom’s cut-off point, as high if the score was between 80 and 100% (16–20 points), moderate if the score was between 50 and 79% (10–15 points), and low if the score was less than 50% (< 10 points). Therefore, sixteen or more points were classified as high knowledge, between ten and fifteen points were rated as moderate knowledge, and fewer than ten points were regarded as low knowledge. We also used five questions to assess participants’ attitudes towards nutrition and applied a Likert scale of Zero-Disagree and One-Agree to grade. Therefore, those who had three or more scores were graded as having a good attitude towards nutrition.

The nutritional status of the participants was analysed using WHO’s BMI formula: [weight (kg)/height (m^2^)] and categorized the outcome into; underweight < 18.5kg/m^2^, normal 18.50–24.99kg/m^2^, overweight ≥ 25–29.99kg/m^2^, and obese ≥ 30kg/m^2^).

### 2.7 Ethical issues

The study protocol was appraised and certified by the Ghana Health Service Ethics Review Committee (GHS-ERC 152/15/17). Permission to conduct the study in the jurisdiction was also sought from the Tema Metro Health Directorate. Besides, written informed consent was obtained from participants above 18 years at their home after the study processes have been explained to them. Those below 18 years, assent and informed consent were also obtained from them and guardians at home after the study processes have been explained to them. Confidentiality, privacy and anonymity were also assured. We obtained the consents by approaching them after attending sessions and were leaving the treatment centres. After explaining the study and they expressed interest to participate in the study, we collected their contact information and trace them to their home for further discussions and consent processes based on the date and time given to us.

## 3. Results

### 3.1 Background characteristics of participants

There were one hundred and forty-six (146) TB patients registered and receiving treatment in health facilities in the study area. The mean age of the participants was 37.2 years (SD ± 3.4), with almost half of them (45.9%) between 20–39 years. Over half (56.2%) of participants were males, 53.4% were legally married, and 55.5% attained a basic level of formal education. The comparative majority (36.3%) of the participants were Akans. About 82.9% were Christians, 61.0% had water closet toilet facilities in their homes, while 10.9% did not have any toilet facility in their homes and, therefore, resorted to open defecation. Majority (61.6%) of the participants were employed in the informal sector (self-employed), while 28.1% were unemployed. About 9.6% had ever smoked cigarette, out of this 35.7% were still smoking at the time of the study, and 66.4% had ever taken alcohol. Also, 59.6% had been on treatment for more than three months, while 67.8% had ever been counselled on nutrition (Table 1).

**Table 1:** Background characteristics of participants.

Variables		Frequency	Percentage
**Sex**	Male	82	56.2
	Female	64	43.8
**Age group (years)**	<20	18	12.3
	20–39	67	45.9
	40–59	46	31.5
	≥ 60	15	10.3
**Ethnicity**	Akan	53	36.3
	Ga	52	35.6
	Ewe	18	12.3
	Krobo	23	15.8
**Religion**	Christianity	121	82.9
	Islam	16	10.9
	Traditional	9	6.2
**Marital status**	Never married	49	33.6
	Legally married	78	53.4
	Cohabiting	13	8.9
	Divorced	6	4.1
**Highest Educational level**	None	20	13.7
	Basic	81	55.5
	Secondary	45	30.8
**Household toilet facility**	Water Closet (WC)	89	61.0
	Pit latrine	41	28.1
	Open defaecation	16	10.9
**Employment status**	Unemployed	41	28.1
	Informal (self-employ)	90	61.6
	Formal (government/company employee)	15	10.3
**Tobacco (cigarette) smoking status**	Never smoked cigarette	132	90.4
	Ever smoked cigarette	14	9.6
**If ever smoked, are you still smoking cigarette**	No	9	64.3
	Yes	5	35.7
**Alcohol consumption status**	Never consumed	49	33.6
	Ever consumed	97	66.4
**Still taken alcohol (n = 97)**	No	32	33.0
	Yes	65	67.0
**Number of months on treatment**	≤ 3 months	59	40.4
	> 3 months	87	59.6
**Received nutritional counselling**	No	47	32.2
	Yes	99	67.8

### 3.2 Nutritional status of participants

About four in ten (41.1%) of TB patients had normal nutritional status, while 39.7% were underweight, 14.4% were overweight, and 4.8% were obese (Table 2).

**Table 2:** Nutritional status of participants.

Nutritional status	Frequency	Percentage
Underweight	58	39.7
Normal	60	41.1
Overweight	21	14.4
Obese	7	4.8

**Fig 1:**
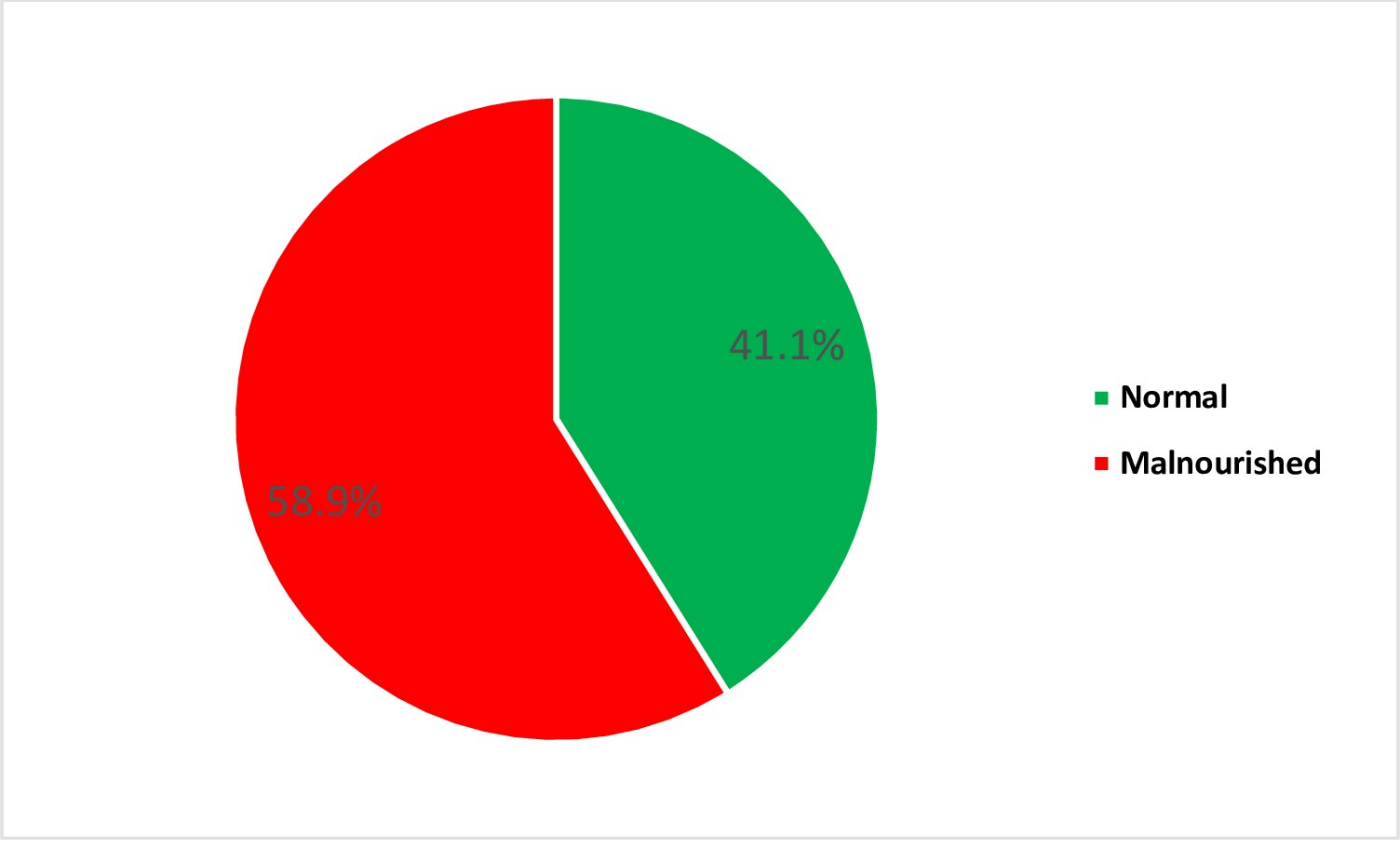
Nutritional status of TB patients.

When the nutritional status was grouped into; normal and malnourished (underweight/overweight/obese), the results showed that 58.9% of the participants were malnourished (Fig 1).

### 3.3 Nutritional knowledge among TB patients

**Fig 2:**
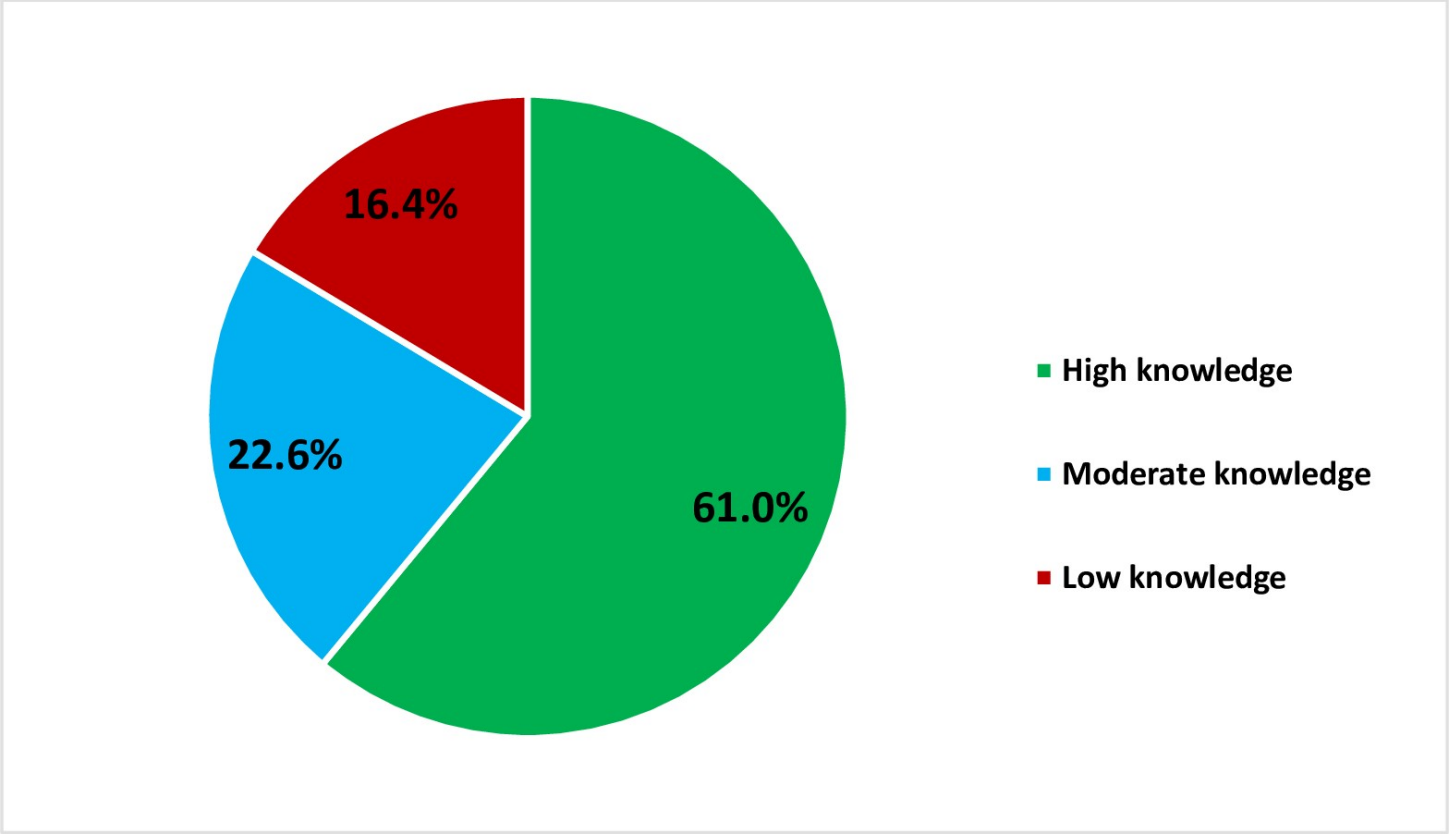
Nutritional knowledge among TB patients.

The results showed that 61.0% of the TB patients had high nutritional knowledge, while 22.6% and 16.4% of them had moderate and low knowledge respectively (Fig 2).

### 3.4 Attitude of TB patients towards nutrition

**Fig 3:**
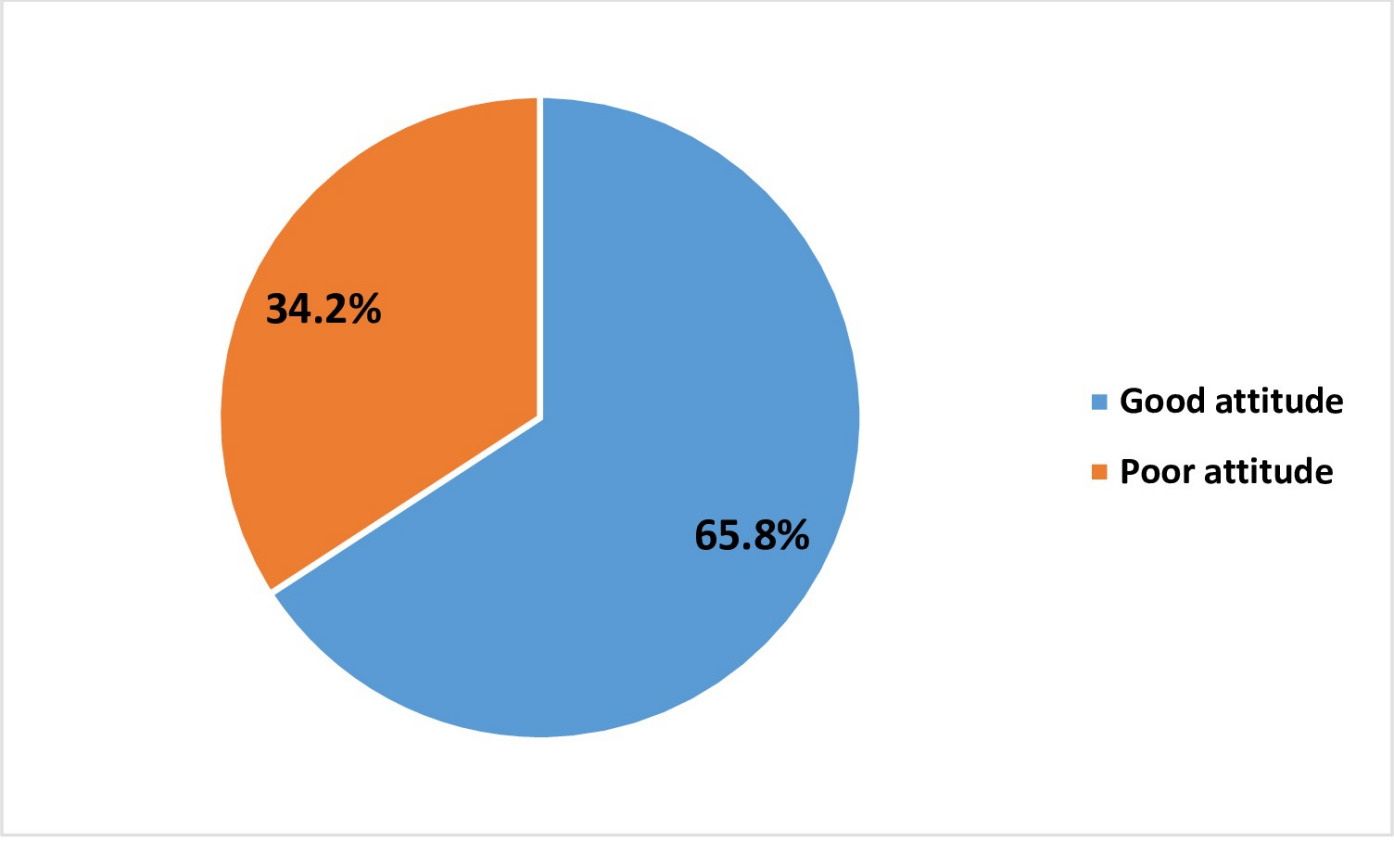
Attitude of TB patients towards nutrition.

The results showed that 34.2% of TB patients have a poor attitude towards nutrition, while 65.8% have a good attitude (Fig 3).

### 3.5 Associations between normal nutritional status and the predictor variables

The bivariate analysis showed statistically significant associations between normal nutritional status and age of participants (*p* = 0.041), educational level (*p* = 0.036), employment status (*p* = 0.019), alcohol intake (*p* = 0.031), period of time TB patient has been on treatment (*p* = 0.021), and attitude towards nutrition (*p* = 0.028). Additionally, when variables that were having associations with normal nutritional status from the bivariate analysis were tested for confounding effects through multiple logistic regression analysis, it confirmed the associations and indicated that TB patients between 20–39 years (AOR: 0.74, 95%CI: 0.02–0.81, *p* = 0.022), 40–59 years (AOR: 0.47, 95%CI: 0.98–0.93, *p* = 0.041), and ≥ 60 years (AOR: 0.19, 95%CI: 0.08–0.93, *p* = 0.039) were less likely to have normal nutritional status than TB patients who were below 20 years. Also, TB patients who had basic education (AOR: 2.46, 95%CI: 2.07–3.86, *p* = 0.031) and secondary education (AOR: 4.21, 95%CI: 2.33–9.49, *p* = 0.026) were more likely to have normal nutritional status than those who did not go to school. TB patients who were employed in the informal (AOR: 2.81, 95%CI: 2.39–9.08, *p* = 0.021) and formal sectors (AOR: 3.77, 95%CI: 3.11–6.89, *p* = 0.014) were more likely to have normal nutritional status than those who were unemployed. Again, TB patients who have ever or are currently taken alcohol (AOR: 0.44, 95%CI: 0.24–0.87, *p* = 0.022) were less likely to have normal nutritional status than those who have never taken alcohol; TB patients who have been on TB treatment for more than 3 months (AOR: 4.01, 95%CI: 1.66–6.33, *p* = 0.018) were more likely to have normal nutritional status compare to those on treatment for three or less months, TB patients who had poor attitude towards nutrition (AOR: 0.49, 95%CI: 0.20–0.71, *p* = .021) were less likely to have normal nutrition status compare to those who had good attitude towards nutrition (Table 3).

**Table 3:** Associations between nutritional status and the predictor variables.

Variables	Nutritional status		COR (95% CI) *p-*value		AOR (95% CI) *p*-value
	Normal	Abnormal			
	60 (41.1%)	86 (58.9%)			
**Sex**					
Male	39 (47.6)	43 (52.4)	1	0.241	
Female	21 (32.8)	43 (67.2)	0.63 (0.25–1.57)		
**Age group (years)**					
<20	15 (83.3)	3 (16.7)	1	**0.041**	1
20–39	40 (59.7)	27 (40.3)	0.65 (0.01–0.79)		0.74 (0.02–0.81) **0.022**
40–59	19 (41.3)	27 (58.7)	0.53 (0.13–0.69)		0.47 (0.30–0.93) **0.041**
≥ 60	12 (80.0)	3 (20.0)	0.82 (0.09–0.98)		0.19 (0.08–0.93) **0.039**
**Ethnicity**					
Akan	23 (43.4)	30 (56.6)	1	0.075	
Ga	18 (34.6)	34 (65.4)	0.17 (0.14–3.21)		
Ewe	9 (50.0)	9 (50.0)	1.68 (0.32–7.08)		
Krobo	10 (43.5)	13 (56.5)	0.89 (0.22–3.56)		
**Religion**					
Christianity	53 (43.8)	68 (56.2)	1	0.067	
Islam	4 (25.0)	12 (75.0)	0.64 (0.09–4.48)		
Traditional	3 (33.3)	6 (66.7)	0.66 (0.12–4.51)		
**Marital status**					
Never married	21 (42.9)	28 (57.1)	1	0.926	
Legally married	32 (41.0)	46 (59.0)	0.54 (0.19–1.57)		
Cohabiting	4 (30.8)	9 (69.2)	0.44 (0.26–3.94)		
Divorced	3 (50.0)	3 (50.0)	1.85 (0.01–8.27)		
**Highest Educational level**					
None	7 (35.0)	13 (65.0)	1	**0.036**	1
Basic	31 (38.3)	50 (61.7)	2.55 (2.09–3.67)		2.46 (2.07–3.86) **0.031**
Secondary	22 (48.9)	23 (51.1)	3.67 (1.25–9.71)		4.21 (2.33–9.49) **0.026**
**Household toilet facility**					
Water Closet	39 (43.8)	50 (56.2)	1	0.529	
Pit latrine	16 (39.0)	25 (61.0)	0.72 (0.24–2.16)		
Open defaecation	5 (31.3)	11 (68.7)	0.26 (0.21–7.62)		
**Employment status**					
Unemployed	19 (46.3)	22 (53.7)	1	**0.019**	1
Informal	36 (40.0)	54 (60.0)	1.66 (1.22–3.57)		2.81 (2.39–9.08) **0.021**
Formal	5 (33.3)	10 (66.7)	2.53 (2.52–3.61)		3.77 (3.11–6.89) **0.014**
**Smoking status**					
Never smoked	53 (40.2)	79 (59.8)	1	0.301	
Ever/currently smoked	7 (50.0)	7 (50.0)	2.05 (0.39–2.79)		
**Alcohol intake**					
Never taken	32 (65.3)	17 (34.7)	1	**0.031**	1
Ever/currently taken	28 (28.9)	69 (71.1)	0.47 (0.17–0.52)		0.44 (0.24–0.87) **0.022**
**Length of being on treatment**					
≤ 3 months	18 (30.5)	41 (69.5)	1	**0.021**	1
> 3 months	42 (48.3)	45 (51.7)	3.64 (2.97–8.73)		4.01 (1.66–6.33) **0.018**
**Received nutritional counselling**					
No	21 (44.7)	26 (55.3)	1	0.613	
Yes	39 (39.4)	60 (60.6)	0.68 (0.37–2.92)		
**Nutrition-related knowledge**					
High knowledge	43 (48.3)	46 (51.7)	1	0.626	
Low/moderate	17 (29.8)	40 (70.2)	0.31 (0.15–3.87)		
**Attitude towards nutrition**					
Good attitude	41 (42.7)	55 (57.3)	1	**0.028**	1
Poor attitude	19 (38.0)	31 (62.0)	0.52 (0.21–0.78)		0.49 (0.20–0.71) **0.021**

## 4. Discussion

The study revealed that 58.9% of the tuberculosis (TB) patients were malnourished, 61.0% of them had high knowledge of nutrition, and 34.2% of them have poor attitude towards nutrition.

Evidence has shown that malnourished tuberculosis patients have delayed recovery and higher mortality rates than their counterparts who are well-nourished because under-nutrition can adversely affect TB treatment outcomes [12]. However, the nutritional status of patients is expected to improve during tuberculosis treatment [13]. The study showed that out of TB patients who were malnourished, 39.7, 14.4, and 4.8% were underweight, overweight, and obese, respectively and revealed that 69.5% of those on treatment for three or fewer months were malnourished, compared to 51.7% of those on medication for over three months, and further indicated that patients on treatment for more than three months were more likely to be well-nourish than those on treatment for three or fewer months. This finding disagrees with the Langsa and Pokhara studies [14,15]. The low rate of malnutrition among patient on treatment for over three months revealed in this study may not be surprising because there is a synergistic relationship between nutrition and infection. For instance, WHO’s monograph on interactions of nutrition and disease has provided enough evidence for the role of infections in triggering malnutrition [16]. Hence it is expected that once the severity of tuberculosis starts to decline, then a patient’s nutritional status will begin to improve because the person food intake and nutrient absorption will enhance, while nutrient depletion will reduce [17].

People who have employment guarantee are better resourced to be able to afford nutritious food compare to those who are not, with evidence shown that families benefited from the Mahatma Gandhi National Rural Employment programme were less likely to have wasted and underweight infants than households that did not benefit from the programme [18], this finding agrees with the present study, which showed that employment status was associated with normal nutritional status of TB patients and indicated that those involved in formal and informal jobs were more likely to be well-nourished than those who have no jobs.

Education offers people the opportunity to be able to read and understand nutritional issues better. A study reported an association between education level and nutritional status and indicates that children whose mothers have a secondary or higher education have a lower risk of childhood stunting, underweight, and wasting compare with children whose mothers did not go to school [19]. This finding agrees with the present study, which showed that TB patients who have basic and secondary education were more likely to have normal nutritional status compare with patients who did not go to school. Another study also reported that education is strongly associated with better health outcomes in both developed and developing countries [20].

Malnutrition has been scientifically associated with older age [21] and agrees with the current study, which revealed that as one grows, the chances of having good nutritional outcomes decreases. The results showed that TB patients between the ages of 20–39, 40–59, and ≥ 60 years were less likely to have normal nutritional status, compare with those below 20 years. However, the study in Northwest Ethiopia disagrees with these findings [22].

Most alcohol addicts are malnourished because they may ingest too little nutrients or because alcohol and its metabolism thwart the body from appropriately absorb, digest, and use essential nutrients. Consequently, drunkards often experience nutrient deficiencies [23]. Again, irregular feeding habits have been related to heavy alcohol intake and indicated that irregular feeding habits among alcoholics lead to malnutrition [24]. The results of the present study agree with these findings and indicated that TB patients who have ever or currently taken alcohol were less likely to have normal nutritional status.

Though knowledge of nutrition may affect the nutritional status of people, the study did not establish any association between nutritional status and knowledge. Yet, the study revealed that 39.0% of the participants have inadequate knowledge of nutrition, which differs from Tanchen Chest Hospital and Gansu province studies [25,26]. Although nutritional counselling is known to cause behaviour change [27], however, this study showed that only 67.8% of the participants were counselled on nutrition while 65.8% of them have good attitudes towards nutrition, and further indicated that participants who have poor attitudes towards nutrition were less likely to be well-nourished, compare to those who have good attitudes towards nutrition. The finding agrees with the India and Nigeria studies [27,28], but disagrees with the study among PLHIV [11].

### 4.1 Study limitations

Nutrition counselling sessions participants have received at the time of the study could influence their nutritional knowledge and status outcomes. However, the study could not assess the number of nutrition counselling sessions each participant received. Also, the quantity of cigarette (tobacco) smoking per day could affect the study outcome, but the study omitted that variable. Notwithstanding, we believe that the limitation cannot invalidate the findings of the study.

## 5. Conclusion

There was reasonable nutrition-related knowledge, and attitude towards nutrition among TB patients. However, this did not translate into high nutritional status. This is despite the fact that nutrition counselling and education have been argued to bring about behaviour change.

### 5.1 Recommendations

1. The Tema Metropolitan Health Directorate, in collaboration with the National TB Control Program, should put in measures to ensure prompt diagnosis and treatment of TB cases, with nutritional support to address malnutrition among TB patients through the establishment of food aid programs.
2. National livelihood intervention programs should be expanded to reach TB patients, particularly those who are unemployed.
3. Healthcare workers in the metropolis should intensify education on smoking cessation and avoidance of alcohol intake among TB patients, as well as the general public.
